# Hematopoietic Stem Cell (HSC)-Independent Progenitors Are Susceptible to Mll-Af9-Induced Leukemic Transformation

**DOI:** 10.3390/cancers15143624

**Published:** 2023-07-14

**Authors:** Cristiana Barone, Roberto Orsenigo, Anna Cazzola, Elisabetta D’Errico, Arianna Patelli, Giulia Quattrini, Barbara Vergani, Silvia Bombelli, Sofia De Marco, Cristina D’Orlando, Cristina Bianchi, Biagio Eugenio Leone, Raffaella Meneveri, Andrea Biondi, Giovanni Cazzaniga, Terence Howard Rabbitts, Silvia Brunelli, Emanuele Azzoni

**Affiliations:** 1 School of Medicine and Surgery, University of Milano-Bicocca, 20900 Monza, Italy; cristiana.barone@unimib.it (C.B.); roberto.orsenigo@vhir.org (R.O.);; 2 Pediatrics, Fondazione IRCCS San Gerardo dei Tintori, 20900 Monza, Italy; 3 Centro Tettamanti, IRCCS San Gerardo dei Tintori, 20900 Monza, Italy; 4 Division of Cancer Therapeutics, Institute of Cancer Research, 15 Cotswold Road, Sutton, London SM2 5NG, UK

**Keywords:** infant AML, Mll-Af9, hematopoiesis, hematopoietic stem cell, erythro-myeloid progenitors, cell of origin, CRISPR/Cas9

## Abstract

**Simple Summary:**

Despite important advances in the understanding of the genetics of infant acute myeloid leukemia (AML), it is still unclear why this blood cancer has features that are distinct to adult AML. Infant AML can often have prenatal origins, but the cell of origin has not been conclusively determined. Using transgenic mice, we found that the introduction of the Mll-Af9 inter-chromosomal rearrangement in hematopoietic stem cell (HSC)-independent progenitors results in a transplantable myeloid leukemia. Thus, we hypothesize that the peculiar characteristics of embryonic hematopoiesis might play a key role in determining the disease phenotype and leukemic progression.

**Abstract:**

Infant acute myeloid leukemia (AML) is a heterogeneous disease, genetically distinct from its adult counterpart. Chromosomal translocations involving the *KMT2A* gene (*MLL*) are especially common in affected infants of less than 1 year of age, and are associated with a dismal prognosis. While these rearrangements are likely to arise in utero, the cell of origin has not been conclusively identified. This knowledge could lead to a better understanding of the biology of the disease and support the identification of new therapeutic vulnerabilities. Over the last few years, important progress in understanding the dynamics of fetal hematopoiesis has been made. Several reports have highlighted how hematopoietic stem cells (HSC) provide little contribution to fetal hematopoiesis, which is instead largely sustained by HSC-independent progenitors. Here, we used conditional Cre-Lox transgenic mouse models to engineer the Mll-Af9 translocation in defined subsets of embryonic hematopoietic progenitors. We show that embryonic hematopoiesis is generally permissive for Mll-Af9-induced leukemic transformation. Surprisingly, the selective introduction of Mll-Af9 in HSC-independent progenitors generated a transplantable myeloid leukemia, whereas it did not when introduced in embryonic HSC-derived cells. Ex vivo engineering of the Mll-Af9 rearrangement in HSC-independent progenitors using a CRISPR/Cas9-based approach resulted in the activation of an aberrant myeloid-biased self-renewal program. Overall, our results demonstrate that HSC-independent hematopoietic progenitors represent a permissive environment for Mll-Af9-induced leukemic transformation, and can likely act as cells of origin of infant AML.

## 1. Introduction

Infant leukemias (defined as those with an onset taking place before 12 months of age) are hematological diseases displaying unique characteristics [1,2]. Whereas leukemias are the most common type of childhood cancer, during infancy they are relatively less frequent (5 versus 1.6–1.7 per 100,000) [3,4]. Clinically, childhood and infant leukemias exhibit different features and the latter are usually characterized by a worse prognosis, largely due to resistance to conventional chemotherapy and the lack of targeted approaches [1,3,5]. In childhood, acute lymphoblastic leukemia (ALL) occurs with a significantly higher frequency than acute myeloid leukemia (AML), whereas the incidence rates of ALL and AML are similar in infants [3].

The genetic alterations found in infant leukemia are different to those generally occurring in older children [3,6]. In particular, rearrangements involving the *MLL* (or *KMT2A*) gene, encoding for the histone lysine methyltransferase 2A, are detected in the majority of infant leukemia patients, and specifically in around 75% of ALL and 50% of AML [7,8]. Mutations in genes involved in the RAS pathway, often found as subclonal events, are also more frequent in infants than in children [3,9]. Generally, infant leukemias carry a small number of genetic lesions and MLL rearrangements are known to act as genetic drivers [6,9]. Interestingly, the same specific rearrangements can be found in both infant ALL and AML. One such example is the Mll-Af9 (t9:11) fusion oncogene, which in infants can result in acute leukemias of either myeloid or lymphoid lineage, whereas in adults the same rearrangement is almost invariably associated with AML [10]. It has been suggested that the cell of origin [11] and/or the hematopoietic microenvironment [12] can affect the outcome of Mll-Af9-induced transformation, and therefore dictate the leukemic phenotype and properties. However, a full understanding of the developmental stage-specific origin of infant leukemia is still missing [13]. Obtaining such knowledge will be critical in order to identify fetal specific cellular and/or environmental clues affecting leukemic biology, and in turn will help in developing better preclinical models and will lead to the identification of targetable vulnerabilities.

The large majority of infant leukemias arise in utero [4,7]. Several efforts have been made in the preclinical modeling of Mll-rearranged leukemias with prenatal origin, and the general consensus is that embryonic-derived leukemias have distinct features compared to those initiated postnatally [3]. When the Mll-Af9 translocation was introduced into fetal hematopoietic stem cells (HSCs) with a knockin model, fetal HSCs showed an increased latency compared to bone marrow HSCs [14]. However, these effects may be context- and model-dependent, as human cord blood CD34+ hematopoietic stem/progenitor cells (HSPCs) were instead easier to immortalize than adult HSPCs [11]. Notably, in both humans and mice, the introduction of Mll-Af9 in fetal HSPCs yielded leukemias of both myeloid and lymphoid lineages, paralleling what happens in patients [11,14]. Other translocations seen in infant AML, such as Mll-Enl, behave more aggressively when induced in fetal or neonatal HSPCs [15,16].

Thus, a relatively new concept of “layered leukemogenicity” has emerged over the last few years, which is trying to link the age-dependent features of leukemias with the peculiar characteristics of embryonic and fetal hematopoietic development [13]. Embryonic hematopoiesis is a remarkably complex process which takes place in several consecutive waves [17,18], and our understanding of it is still incomplete. In recent years, a new paradigm has emerged in the field, highlighting how HSCs do not primarily contribute to fetal hematopoiesis, which is instead primarily sustained by HSC-independent progenitors [19,20,21]. The fetal liver (FL) has long been recognized as the major hematopoietic organ during the second half of gestation, and it is perceived as an important site of HSC expansion [22]. However, a recent study challenged this long-standing view by showing that HSCs fated to adult life do not significantly expand in the FL but are instead kept quiescent [23], which is in agreement with HSC-independent progenitors playing a major role in pre-natal hematopoiesis. At the same time, the FL microenvironment can function as a hematopoietic differentiation hub for fetal-specific progenitors which emerge in other locations as a “predefined” hierarchy [21,24]. These results evidence a previously unrecognized complexity in FL hematopoiesis, which, as a result, can impact our understanding of leukemias with prenatal origin, and, consequently, their modeling.

Most experimental strategies used so far to model fetal-derived leukemias have considered FL HSPCs “as a whole”; however, HSPCs in the mid-gestation FL are highly heterogeneous and can have various origins and fates. Therefore, in order to generate faithful preclinical models of childhood and infant leukemias, we need different approaches which should take into account the complexity of embryonic and fetal hematopoiesis. To date, it is unclear whether HSC-independent progenitors may be permissive for Mll rearrangement-mediated leukemic transformation, and thus could act as cells of origin for infant AML. Here, we aimed at gaining a better understanding of the ontogeny of infant Mll-Af9-rearranged leukemia. To this scope, we employed different genetic strategies in order to introduce the Mll-Af9 translocation in defined subsets of embryonic HSPCs, including HSC-independent progenitors.

## 2. Materials and Methods

### 2.1. Mouse Strains

All mouse strains used in this study have been previously described. To obtain mice bearing the Mll-Af9 translocation under constitutive expression of the Cre recombinase in embryonic hematopoietic progenitors, *Tie2-Cre* transgenic mice [25] were crossed with *Mll^loxP^::Af9^loxP^* “translocator” mice [26,27]. For the generation of a mouse model in which the Mll-Af9 translocation could be induced at specific time-points in embryogenesis, we used *Cdh5-CreER^T2^* mice [28,29,30]. For the induction of the Mll-Af9 translocation using the CRISPR/Cas9 system, *Cdh5-CreER^T2^* mice were crossed with *Rosa26-LSL-Cas9-EGFP* knockin mice [31]. For lineage tracing experiments, *Cdh5-CreER^T2^* mice were crossed with *Rosa26-LSL-tdTomato* or *Rosa26-LSL-zsGreen* mice [32].

To define the gestational age of mouse embryos, mice were mated in the evening and the presence of a vaginal plug the following morning was defined as embryonic day(E)0.5. Time-specific Cre induction was achieved through an intra-peritoneal (i.p.) injection of 4-hydroxytamoxifen (4-OHT) (37.5 mg/kg) or tamoxifen (75 mg/kg).

All transgenic mouse lines were maintained on a CD45.2 C57BL/6 genetic background, with the exception of females used for timed mating in order to generate adult mice with 4-OHT activation during embryogenesis, which were instead of a C57BL/6/FVB mixed background (F1). For transplantation experiments, CD45.1 C57BL/6 syngeneic mice were used as recipients. In some experiments, donor cells were CD45.1/CD45.2 heterozygotes. Mice were housed with free access to food and water at the San Raffaele Scientific Institute institutional mouse facilities. All experiments were performed in accordance with experimental protocols approved by the local Institutional Animal Care and Use Committees (IACUC).

### 2.2. Polymerase Chain Reaction (PCR) for Mll-Af9 Detection and Sequencing

The detection of the Mll-Af9 translocation induced with the Cre-loxP system was performed via nested PCR to increase the amplification specificity, as previously reported [26]. DNA was obtained from the fetal liver (FL) or adult bone marrow (BM) of transgenic and wild type embryos. For both the Cre-loxP- and CRISPR/Cas9-induced translocation, a HotStarTaq Master mix kit (Qiagen, Hilden, Germany) was used. PCR primers used for Mll-Af9 detection are reported in Table S1.

Confirmation of the correct inter-chromosomal translocation after CRISPR/Cas9 editing was achieved through Sanger sequencing.

### 2.3. Flow Cytometry and Hematological Analysis

Flow cytometry analysis was performed as previously described [33]. Bleedings were performed from the mouse tail vein every 8 weeks (unperturbed model) or every 4 weeks (transplants). BM cells were extracted from the femur and tibia. Briefly, samples were stained and analyzed in CMF (Calcium/Magnesium-Free PBS, 10% FBS, 5% Pen/Strep, 2 mM EDTA), incubated for 15 min with 0.5 mg/mL Fc block (1:500) (BD Biosciences, Franklin Lakes, NJ, USA) and labeled with a combination of PE-conjugated rat anti-CD45 (1:200) (BD Biosciences), PE-conjugated rat anti-CD45.1 (1:200) (BD Biosciences), FITC-conjugated rat anti-CD45.2 (1:400) (BD Biosciences), PE-Cy7-conjugated rat anti-CD11b (1:200) (BD Biosciences), APC-Cy7-conjugated rat anti-B220 (1:200) (BioLegend, San Diego, CA, USA), PE-Cy5-conjugated rat anti-CD3e (1:100) (BioLegend), and APC-conjugated rat anti-Gr1 (1:200) (BioLegend). Appropriate fluorescence gating parameters were established with compensation beads (BD Biosciences), unstained, and fluorescence-minus-one (FMO) staining. In all experiments, doublets were gated out using pulse geometry gates (FSC-H versus FSC-A and SSC-H versus SSC-A), whereas dead cells were excluded using Hoechst 33258 (Hellobio, Bristol, UK). Single-cell suspensions were analyzed using a Fortessa X-20 analyzer (BD Biosciences). FlowJo software v10 (FlowJo/BD, Ashland, OR, USA) was used for subsequent analyses. For hematological analyses, peripheral blood was analyzed using an ABX Pentra 60 C+ analyzer (HORIBA Medical, Kyoto, Japan).

### 2.4. Primary and Secondary Transplants

For transplantation experiments, syngeneic C57BL/6 (CD45.1) recipient mice were lethally irradiated (9 Gy, split dose) before the intra-venous transplantation of 2 × 10^6^ unfractionated E14.5 FL (primary) or adult BM cells (secondary) from *Tie2-Cre::Mll^loxP^::Af9^loxP^* and *Cdh5-CreER^T2^*::*Mll^loxP^::Af9^loxP^* (4-OHT at E8.5 or E10.5). Donor-derived chimerism and hematological parameters within donor cells were determined using flow cytometry in peripheral blood (PB) every 4 weeks post-transplantation, and in PB and BM at 8 (*Tie2-Cre::Mll^loxP^::Af9^loxP^*), 28 (*Cdh5-CreER^T2^*::*Mll^loxP^::Af9^loxP^* primary transplants), and 12 (*Cdh5-CreER^T2^*::*Mll^loxP^::Af9^loxP^* secondary transplants) weeks post-transplantation.

For the transplantation experiments of CRISPR/Cas9-edited cells, C57BL/6 (CD45.1) recipient mice were sub-lethally irradiated (4.5 Gy, single dose) before the intra-venous transplantation of 2–4 × 10^5^ E16.5 CD45+ c-Kit+ GFP+ FL cells from *Cdh5-CreER^T2^::R26-LSL-Cas9-GFP* embryos (4-OHT at E8.5).

### 2.5. Histological Analysis

The spleen, liver, and lungs were collected from wild type and leukemic mice. Tissue samples were fixed in a 4% solution of paraformaldehyde (PFA) in PBS, dehydrated with ethanol, cleared with xylenes, and embedded in paraffin. Then, 5 mm organ sections were cut on a rotary microtome and stained with hematoxylin and eosin to assess the presence of extramedullary leukemic infiltrates. The size and weight of spleens were also recorded.

### 2.6. Fluorescence-Activated Cell Sorting (FACS) for the Isolation of HSC-Independent Hematopoietic Progenitors

For FACS, E14.5 FL from *Cdh5-CreER^T2^::R26-LSL-Cas9-GFP* were dissected and enzymatically digested with collagenase type I (Sigma-Aldrich, St.Louis, MO, USA) 0.12% (*w*/*v*), followed by mechanical dissociation via pipetting. Cells were incubated with Fc block (1:500) (BD Biosciences), PE-conjugated rat anti-CD45 (1:200) (BD Biosciences), and PE-Cy7-conjugated rat anti-CD117 (c-Kit, 1:200) (BD Biosciences). Live single CD45+ c-Kit+ GFP+ cells were isolated using a MoFlo Astrios Cell Sorter equipped with Summit software version 6.1 (Beckman Coulter, Brea, CA, USA).

### 2.7. Single Guide RNAs (sgRNA) Design and Nucleofection

sgRNAs were purchased from Synthego (Redwood City, CA, USA) and designed using the bioinformatic tools GT-Scan [34] and ATUM gRNA Design tool (ATUM, Newark, CA, USA) to target sites within intron 10 and intron 8 of the Mll and Af9 genes, respectively. The best guides were chosen based on the highest cutting score and the minimum potential off-targets with sequences containing up to 3 mismatches. The sgRNA sequences are Mll: 5′-AGGTCTGTCTTCTGCTACGC-3′; Af9: 5′-GTTGCACTTTCGGAATGTGT-3′.

The nucleofection of target cells was performed with an Amaxa Nucleofector 4D (Lonza, Basel, Switzerland), following the manufacturer’s protocol. The program of nucleofection used here was CM-137. Nucleofected cells were then transferred on fresh medium and co-cultured with OP9 stromal cells for a maximum of 7 days (described below).

### 2.8. CFU-C Assays

Sorted Cas9-expressing hematopoietic progenitors (CD45+ c-Kit+ GFP+) nucleofected with both sgRNAs against Mll and Af9 were cultured with MethoCult GF M3434 (Stem Cell Technologies, Vancouver, BC, Canada), as per the manufacturer’s instructions, to give rise to CFU-E, CFU-G/M/GM, and CFU-GEMM. The number and identity of colonies were scored after 7 days, after which replating with an equal number of starting cells was performed.

### 2.9. Cell Cultures

All cell cultures were grown at 37 °C, 5% CO_2_. Hematopoietic progenitor cells isolated from E14.5 FL were co-cultured with OP9 cells and maintained in Iscove’s Modified Dulbecco’s Medium Glutamax (IMDM) (GIBCO/ThermoFisher, Paisley, Scotland, UK) supplemented with 10% Fetal Bovine serum (FBS) (HyClone, Logan, UT, USA), 1% Bovine Serum Albumin (BSA), 1% Pen/Strep, 50 ng/mL SCF, 50 ng/mL IL-3, and 50 ng/mL FLT-3L for a maximum of 7 days. All cytokines were purchased from PeproTech (Cranbury, NJ, USA).

### 2.10. Statistical Analysis

No specific randomization method was used in the present study. Experimental animals were allocated to groups according to their genotype. No specific methods were used for blinding. In general, samples were collected from mice by one individual and then processed and analyzed by different individuals, at a time during which the genotypes or experimental conditions of each sample were not known. To determine the level of significance, an unpaired Student’s *t*-test with Welch correction was used through GraphPad Prism software version 9.4.1. *p* < 0.05 was considered statistically significant, and the level of significance is indicated by asterisks: * *p* < 0.05; ** *p* < 0.01; *** *p* < 0.001; **** *p* < 0.0001.

## 3. Results

### 3.1. In Vivo Introduction of Mll-Af9 Inter-Chromosomal Translocation in Embryonic HSPCs Results in a Transplantable Myeloproliferative Disease

To generate a model in which the Mll-Af9 translocation is introduced in vivo in embryonic HSPCs, we took advantage of the Mll-Af9 “translocator” mouse model, in which *loxP* sites are inserted in *Mll* and *Af9* genes in positions corresponding to the breakpoints observed in patients [26]. In the presence of Cre recombinase, in vivo inter-chromosomal recombination events are induced, sufficient to cause transplantable myeloid leukemia provided the cellular context is permissive [27].

**Figure 1:**
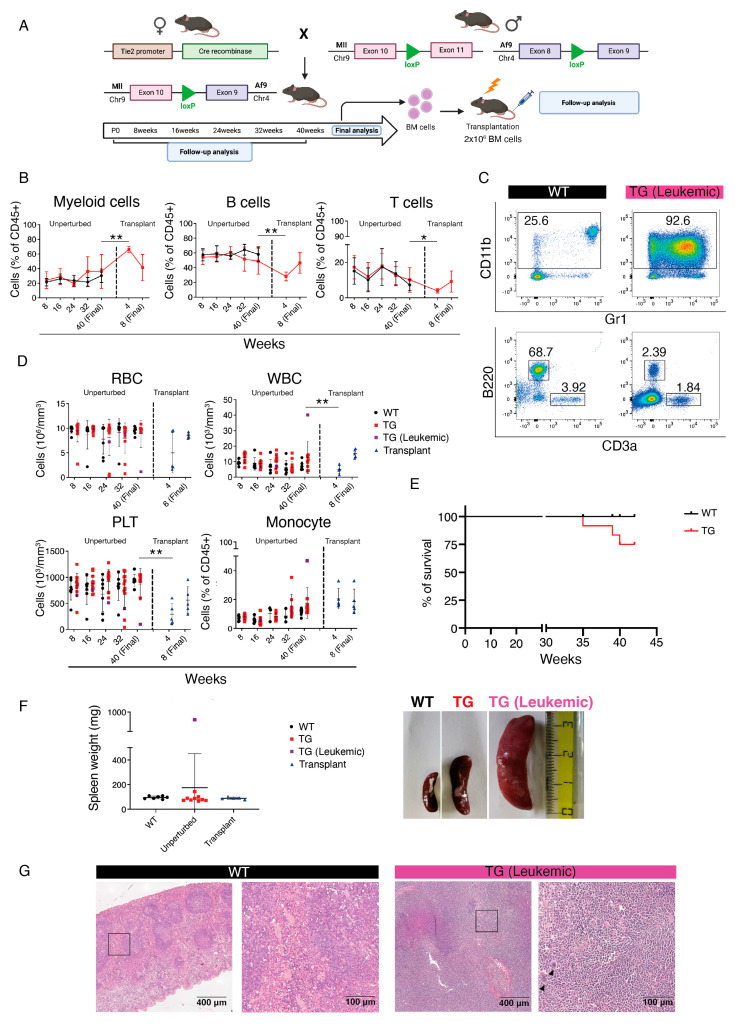
Generation and analysis of *Tie2-Cre::Mll^loxP^*::*Af9^loxP^*mice. (**A**) Schematic of crossing strategy and analyses of *Tie2-Cre::Mll^loxP^::Af9^loxP^* mice. (**B**) Quantification of flow cytometric analysis displayed in (**C**) of myeloid (left), B (center), and T cells (right) in *Tie2-Cre::Mll^loxP^::Af9^loxP^* (TG, red) or wild-type (WT, black) peripheral blood (PB) in unperturbed and transplant settings. Data are shown as mean ± standard deviation (SD). * *p* < 0.05, ** *p* < 0.01. Indicated statistical comparisons were made between transgenic (TG) mice at the indicated time points. (**C**) Representative flow cytometric analysis of PB cells in unperturbed WT (left) or leukemic *Tie2-Cre::Mll^loxP^::Af9^loxP^* (TG, right) adult mice at 40 weeks. (**D**) Red blood cell (RBC, top left), white blood cell (WBC, top right), platelets (PLT, bottom left) absolute counts, and monocyte percentage (bottom right) in *Tie2-Cre::Mll^loxP^::Af9^loxP^* (TG, red or leukemic TG, purple) or WT mice (black) in unperturbed and transplant settings. Data are mean ± SD. ** *p* < 0.01. (**E**) Kaplan–Meier survival curve of WT and TG (*Tie2-Cre::Mll^loxP^::Af9^loxP^)* mice over the course of the analysis. (**F**) (Left) Spleen weight of WT (left), unperturbed TG (center), and transplanted (right) mice. The leukemic TG mouse that was analyzed is shown in purple. (Right) Comparison between the spleen size of unperturbed background WT (top), *Tie2-Cre::Mll^loxP^::Af9^loxP^* non-leukemic TG (center), and leukemic TG (right) mice. Data are mean ± SD. (**G**) Spleen histology of unperturbed mice, showing the typical splenic structure in the WT (left) compared to the absence of splenic architecture in the leukemic *Tie2-Cre::Mll^loxP^::Af9^loxP^* (right), with the presence of histiocytic infiltrates (black arrow heads). Boxed areas in left panels of WT and TG (Leukemic) are magnified on the right. Scale bar: 400 μm (left panels), 100 μm (right panels).

Here, we crossed Mll-Af9 translocator mice with *Tie2-Cre* transgenic mice (Figure 1A), which express a constitutive Cre recombinase under the control of the Tie2 promoter, active in endothelial cells from early embryonic stages, including the hemogenic endothelium (HE), a specialized transient cell type present during ontogeny which is the precursor to most blood cells [35]. Hence, genetic targeting with the *Tie2-Cre* allele results in recombination in embryonic hematopoietic progenitor cells, which eventually give rise to the large majority of postnatal blood cells [36].

*Tie2-Cre::Mll^loxP^::Af9^loxP^* mice were born at normal expected Mendelian ratios and we could detect evidence of the Mll-Af9 inter-chromosomal translocation in the bone marrow (BM) of these animals by PCR (Figure S1A). Periodic follow-up by peripheral blood (PB) flow cytometric analysis and blood counts showed signs of overt disease in a few cases out of a cohort of 12 double transgenic mice, in which a large increase in the percentage of myeloid cells and concomitant decrease in B and T lymphocytes was detected already from 32 weeks (Figure 1B,C).

Accordingly, leukocytes, red blood cells (RBC), and platelets (PLT) did not show alterations except in leukemic mice, whereas the percentage of monocytes showed a steady increase in all transgenic mice, apparent from 32 weeks (Figure 1D). Unfortunately, two transgenic mice died before the final analysis, likely of myeloid leukemia (Figure 1E). Mice were sacrificed at 40 weeks, and the spleen of a leukemic mouse, which showed a large increase in myeloid cells, was found to be abnormally enlarged (Figure 1F). A histological analysis found a complete subversion of the splenic architecture composed of white and red pulp, and the presence of large nucleated cells, identifiable as histiocytic infiltrates (arrowheads in Figure 1G). These results showed that the introduction of the Mll-Af9 translocation in embryonic HSPCs could generate a myeloproliferative disease.

To determine whether BM cells from *Tie2-Cre::Mll^loxP^::Af9^loxP^* mice contained cells able to propagate the disease, we performed transplants into lethally irradiated syngeneic recipients (Figure 1A and Figure S1B). We transplanted cells from four different transgenic mice into seven recipients (1 to 2 recipient mice for each donor). Notably, the two recipients transplanted with the BM of the primary mouse showing the overt leukemic phenotype died 14 and 15 days post-transplant, probably due to severe anemia; therefore, they were not included in the subsequent analysis. In all other cases, we could detect a large increase in myeloid cells accompanied by significant reduction in B lymphocytes already from 4 weeks post-transplant (Figure 1B and Figure S1B). Because by 8 weeks post-transplantation recipient mice showed clear signs of suffering with large increases in leukocyte counts and monocytes (Figure 1D) and weight loss [37], these animals were suppressed. These data suggest that the myeloid disease resulting from the introduction of Mll-Af9 in embryonic HSPCs is transplantable.

### 3.2. Transplantation of HSC-Independent Hematopoietic Progenitors Carrying Mll-Af9 Translocation Induces a Myeloid Neoplastic Disease

**Figure 2:**
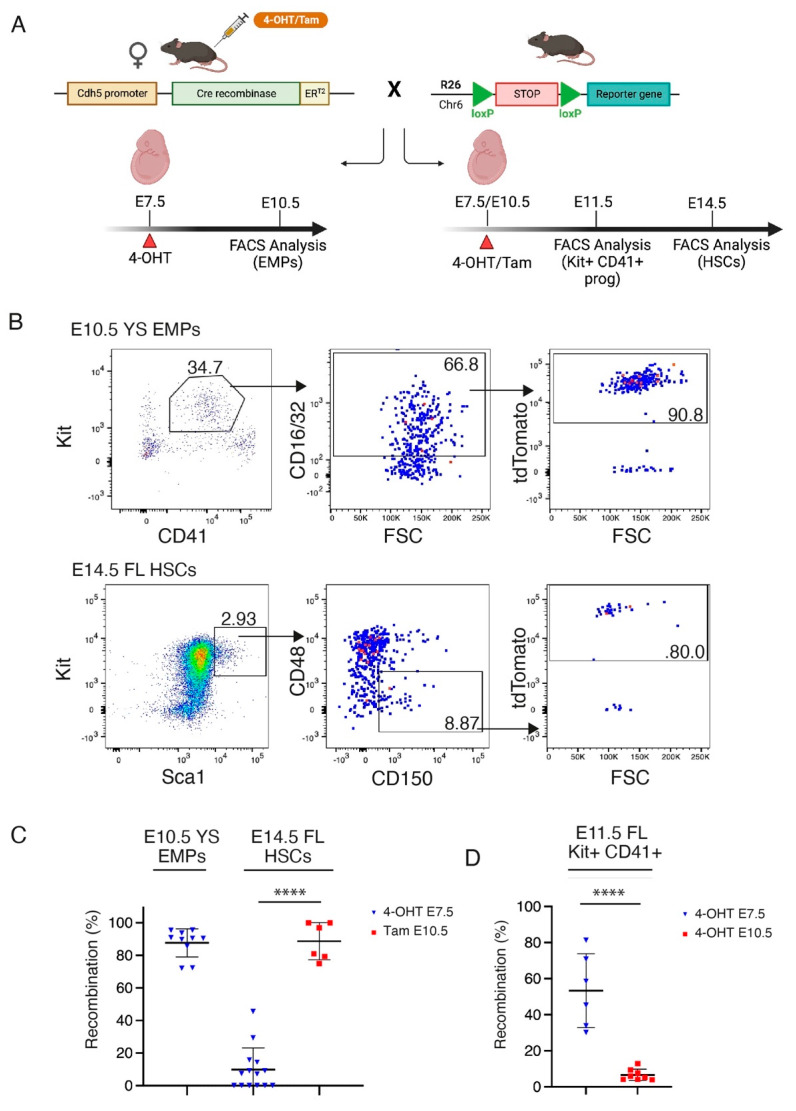
Validation of *Cdh5-CreER^T2^* mice for selective labeling of EMPs and HSCs. (**A**) Schematic of crossing strategy and analyses of *Cdh5-CreER^T2^::R26-tdTomato/zsGreen* mice for lineage tracing analysis. (**B**) Representative flow cytometric analysis of *CreER^T2^::R26-tdTomato* embryos. E10.5 YS EMPs activated with 4-OHT at E7.5 (upper) and E14.5 FL HSCs activated with tamoxifen (Tam) at E10.5 (lower) are shown. (**C**) Quantification of flow cytometric analyses displayed in (**B**), plus analysis of E14.5 FL HSCs activated with 4-OHT at E7.5. Each dot represents a single embryo. (**D**) Flow cytometric analysis of Kit+ CD41+ hematopoietic progenitor cells in *Cdh5-CreER^T2^::R26-tdTomato/zsGreen* E11.5 FL activated with 4-OHT at E7.5 (blue) or E10.5 (red). Each dot represents a single embryo. Data are shown as mean ± SD. **** *p* < 0.0001.

The *Tie2-Cre* allele directs recombination to all subsets of HE, and therefore targets both HSC-independent progenitors (such as erythro-myeloid progenitors, or EMPs) and HSCs [38]. To generate a model that would allow the selective introduction of the Mll-Af9 translocation into HSC-independent progenitors separate from HSCs, we turned to an inducible strategy by employing the *Cdh5-CreER^T2^* model. It was previously shown that the administration of 4-hydroxytamoxifen (4-OHT) at E7.5 and E10.5 in these mice can direct recombination to EMPs and HSCs, respectively [28]. We validated this experimental strategy by performing lineage tracing experiments in which we crossed *Cdh5-CreER^T2^* mice with *R26-LSL-tdTomato* or *R26-LSL-zsGreen* reporters [32] and induced Cre recombination with a single dose of 4-OHT or tamoxifen at E7.5 or E10.5 (Figure 2A). We confirmed that 4-OHT at E7.5 directs recombination to the majority of EMPs in the E10.5 yolk sac (YS) (Figure 2B,C) and in the E11.5 FL (Figure 2D), while largely avoiding recombination in E14.5 FL HSCs (Figure 2B,C). Conversely, E10.5 tamoxifen administration yields recombination in most E14.5 FL HSCs (Figure 2B,C). Notably, E10.5 Cre induction (performed with 4-OHT, chosen over tamoxifen in this particular experiment because of its more rapid metabolization) [39] avoids targeting the recombination to EMPs in the E11.5 FL (Figure 2D).

**Figure 3:**
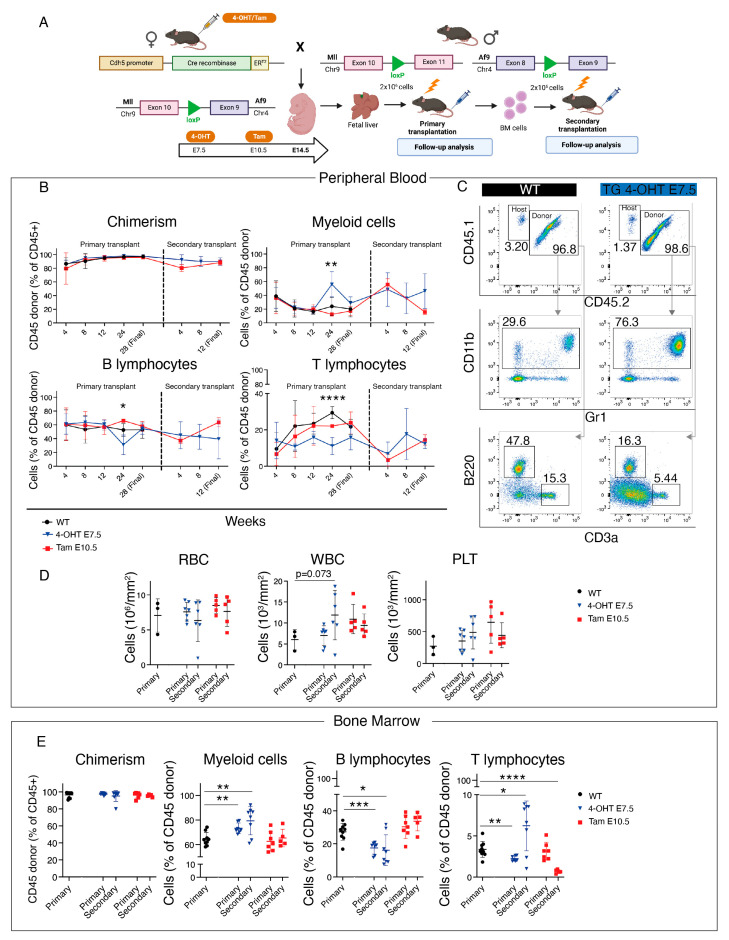
Analysis of *Cdh5-CreER^T2^*::*Mll^loxP^::Af9^loxP^* primary and secondary transplants. (**A**) Schematic of crossing strategy, Cre activation (4-OHT at E7.5 or tamoxifen at E10.5) and analyses of primary fetal liver (FL) and secondary bone marrow (BM) *Cdh5-CreER^T2^::Mll^loxP^::Af9^loxP^* transplants. (**B**) Quantification of PB flow cytometric analysis displayed in (**C**) of donor-derived chimerism (top left), donor myeloid (top right), (**B**) (bottom left) and T lymphocytes (bottom right) in primary and secondary transplant recipients of WT (black) and *Cdh5-CreER^T2^::Mll^loxP^::Af9^loxP^* cells with the administration of 4-OHT at E7.5 (blue) or tamoxifen at E10.5 (red). Data are mean ± SD. ** *p* < 0.01, **** *p* < 0.0001. The statistical significance refers to the comparison of 4-OHT E7.5-activated *Cdh5-CreER^T2^::Mll^loxP^::Af9^loxP^* mice with WT mice. (**C**) Representative flow cytometric analysis of PB chimerism (top), donor myeloid (center), B and T lymphocytes (bottom) in primary transplant recipient mice of WT (left) or 4-OHT E7.5-activated *Cdh5-CreER^T2^::Mll^loxP^::Af9^loxP^* (TG, right) cells. Donor cells are CD45.1+/CD45.2+; host cells are CD45.1+. (**D**) PB, RBC (left), WBC (center), PLT (right) absolute counts of primary and secondary transplant recipient mice of WT (black) and *Cdh5-CreER^T2^::Mll^loxP^::Af9^loxP^* cells with the administration of 4-OHT at E7.5 (blue) or tamoxifen at E10.5 (red). Analysis was performed in 28-week-old mice for primary transplants, and 12-week-old mice for secondary transplants. Data are mean ± SD. (**E**) Quantification of BM flow cytometric analysis of donor-derived chimerism (left), myeloid (center left), B (center right), and T lymphocytes (right) in primary and secondary transplant recipient mice of WT (black) and *Cdh5-CreER^T2^::Mll^loxP^::Af9^loxP^* cells with the administration of 4-OHT at E7.5 (blue) or tamoxifen at E10.5 (red). Data are mean ± SD. * *p* < 0.05, ** *p* < 0.01, *** *p* < 0.001, **** *p* < 0.0001.

**Figure 4:**
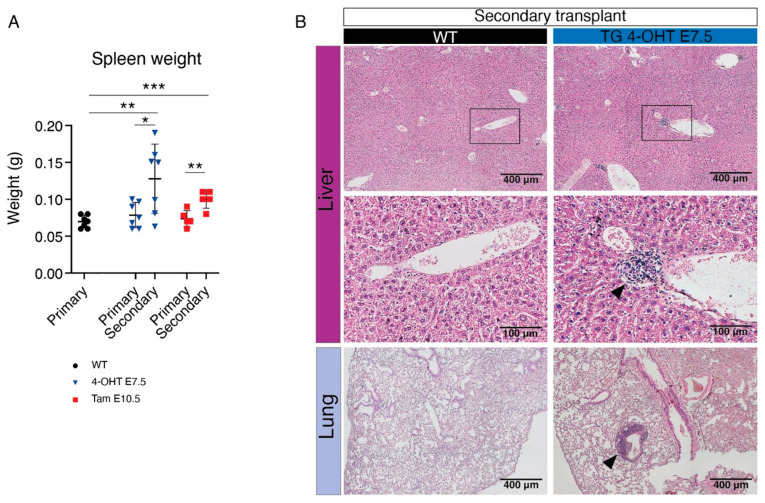
Morphological and histological analysis of extramedullary hematopoiesis in *Cdh5-CreER^T2^*::*Mll^loxP^::Af9^loxP^* transplanted mice. (**A**) Spleen weight of WT (black) and *Cdh5-CreER^T2^::Mll^loxP^::Af9^loxP^* mice with the administration of 4-OHT at E7.5 (blue) or tamoxifen at E10.5 (red) in primary and secondary transplants. Data are mean ± SD. * *p* < 0.05, ** *p* < 0.01, *** *p* < 0.001. (**B**) Histological analysis showing the presence of extramedullary leukocyte infiltration (black arrow heads) in liver (top and middle) and lung (bottom) in 4-OHT E7.5-activated *Cdh5-CreER^T2^::Mll^loxP^::Af9^loxP^* (right) secondary transplants. WT controls (left) show absence of extramedullary infiltration. Middle panels are magnifications of boxed areas shown in top panels. Scale bar: 400 μm (top and bottom panels), 100 μm (magnification, middle panels).

Next, we crossed *Cdh5-CreER^T2^* with *Mll^loxP^::Af9^loxP^* mice and activated Cre recombination with 4-OHT at E7.5 or E10.5 (Figure S2A). Double transgenic mice were born at normal ratios and were followed up for 48 weeks, without evidence of alterations in blood parameters or in the percentage of myeloid cells, B, and T lymphocytes (Figure S2B,C). As with the *Tie2-Cre* constitutive strategy, the leukemic phenotype was more evident after transplantation than in the unperturbed background (Figure 1); in order to conclusively determine whether HSC-independent progenitors are susceptible to Mll-Af9-mediated transformation, we next decided to combine the inducible Cre-Lox strategy with transplantation. We crossed *Cdh5-CreER^T2^* with *Mll^loxP^::Af9^loxP^* lines and activated Cre recombination with 4-OHT at E7.5 or tamoxifen at E10.5 (Figure 3A). For this specific time point, we used tamoxifen as it has a longer half-life than 4-OHT, and therefore yields recombination in a higher percentage of fetal HSCs ([39]; unpublished data). We isolated E14.5 FL from *Cdh5-CreER^T2^*::*Mll^loxP^::Af9^loxP^* and Cre-controls (“WT”), and transplanted cells into lethally irradiated recipients (Figure 3A). The 352-bp PCR band corresponding to the Mll-Af9 translocation was detected in 100% of the transgenic FL samples activated at E7.5, whereas in 50% of those activated at E10.5, probably reflecting the fact that in negative samples the number of cells carrying the translocation was under the threshold level of detection at this stage (Figure S3A,B). In all recipient mice, from 12 weeks post-transplant onwards, PB donor chimerism was high (>90%) (Figure 3B). At 24 weeks post-transplant, we detected a significant increase in the percentage of donor-derived PB myeloid cells with a decrease in B and T lymphocytes only in mice transplanted with FL activated at E7.5 (Figure 3B,C). Therefore, at 28 weeks post-transplant, we sacrificed transplanted mice. Absolute counts of leukocytes, RBC, and PLT did not show significant differences at this time point (Figure 3D), and the spleen sizes of transgenic and control animals were comparable (Figure 4A). BM analysis showed very high donor chimerism in all animals, and, again only with the E7.5 activation, a significant increase in myeloid cells was observed, accompanied by a decrease in B and T lymphocytes (Figure 3E), although this was not mirrored in the PB (Figure 3B).

Next, we performed secondary transplantations using the BM of animals transplanted with transgenic FL cells. Secondary transplanted mice quickly developed signs of disease with prominent leukocytosis in all animals (Figure 3D), which were therefore sacrificed at 12 weeks post-transplant. The Mll-Af9 PCR band was detected in the majority of secondary transplanted animals in both experimental conditions, suggesting that the expansion of cells carrying the translocation had occurred (Figure S3). However, only animals transplanted with cells activated at E7.5 showed a significant increase in myeloid cells in the BM, with a concomitant decrease in B lymphocytes, whereas with the E10.5 activation we observed a slightly increased percentage of B lymphocytes and no change in myeloid cells (Figure 3E). Secondary transplanted mice showed significantly enlarged spleens, particularly those transplanted with cells activated at E7.5 (Figure 4A). A histological examination of the liver and lungs from these animals showed clear signs of extramedullary hematopoiesis in both organs, with leukocyte infiltrates often found adjacent to vessels (Figure 4B).

Taken together, these results support the idea that HSC-independent progenitors, including EMPs, are susceptible to leukemic transformation upon acquisition of the Mll-Af9 inter-chromosomal translocation.

### 3.3. CRISPR/Cas9 Engineering of the Mll-Af9 Translocation in HSC-Independent Hematopoietic Progenitors Triggers an Aberrant Self-Renewal Program

An acute leukemic phenotype was only evident in secondary transplanted mice in which the Mll-Af9 rearrangement was introduced in HSC-independent progenitors, which suggests a long latency, possibly reflecting the low efficiency of the translocator mouse-based approach. It is not clear whether Mll-Af9 can directly affect the self-renewal capacity of HSC-independent progenitors. To answer this question, we decided to employ a CRISPR/Cas9-based approach. Thus, we combined CRISPR/Cas9 knockin mice [31] with an electroporation-based method for the engineering of chromosomal rearrangements [40,41], which we adapted and optimized here for mouse FL cells.

**Figure 5:**
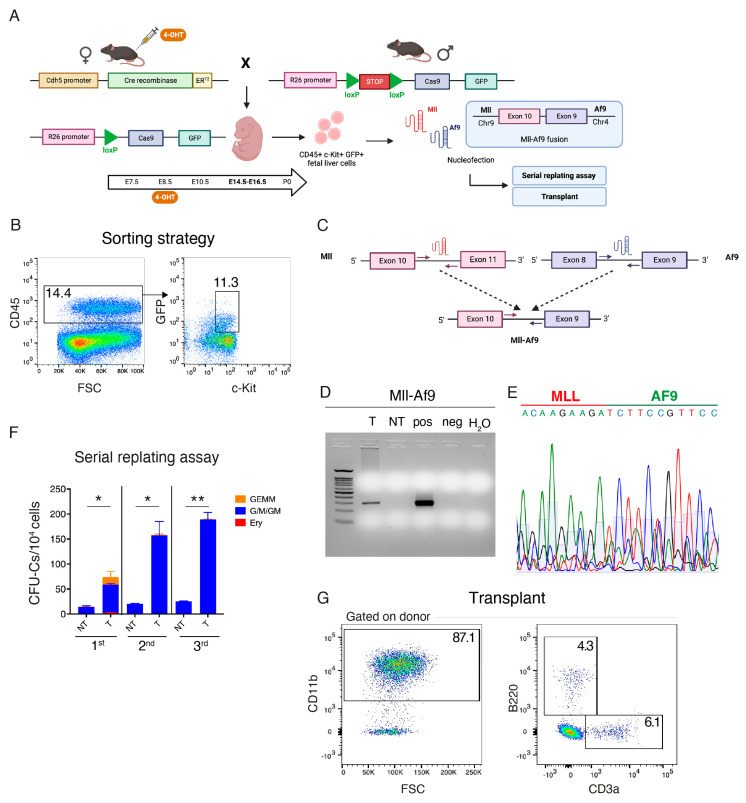
CRISPR/Cas9 engineering of the Mll-Af9 fusion gene in hematopoietic stem cell (HSC)-independent hematopoietic progenitors. (**A**) Experimental schematic of the strategy for the isolation, gene editing, and analysis of fetal HSC-independent progenitors. (**B**) FACS isolation of HSC-independent progenitors. Live CD45+ c-Kit+ GFP+ cells were sorted from the FL of *Cdh5-CreER^T2^::R26-LSL-Cas9-GFP* E14.5 embryos (4-OHT at E8.5). (**C**) sgRNA were designed to target intron 10 of the Mll (red) and intron 8 of the Af9 (blue) genes. When electroporated in combination, the formation of the inter-chromosomal Mll-Af9 translocation is induced. Arrows represent PCR primers used to detect the translocation band. (**D**) Representative agarose gel showing the presence of the Mll-Af9 translocation with an amplicon of the expected size (300 bp) observed in transfected cells (T), and absent in non-transfected cells (NT). Positive (pos) and negative (neg) controls are shown. (**E**) Sanger sequencing confirming the identity of the Mll-Af9 translocation. The variability in the peaks around the breakpoint is due to the sequencing reaction being performed on bulk-edited cells. A = green; C = blue; G = black; T = red. (**F**) Serial replating CFU assay with sorted hematopoietic stem and progenitor cells (HSPCs) transfected with the combination of the sgRNAs against Mll and Af9 genes (T) and non-transfected HSPCs (NT). Colonies were scored after 7 days following plating. Granulocyte/erythroid/monocyte/macrophage (GEMM, orange), granulocyte/macrophage (G/M/GM, blue), and erythroid (Ery, red) colonies were scored. Data are mean ± SD. * *p* < 0.05, ** *p* < 0.01. (**G**) Representative peripheral blood flow cytometric analysis of recipient mice engrafted with Mll-Af9 edited E16.5 FL HSPCs, sampled 4 weeks post-transplantation (n = 2 mice transplanted).

To selectively introduce the Mll-Af9 translocation in HSC-independent hematopoietic progenitors using CRISPR/Cas9, we crossed *Cdh5-CreER^T2^* with *R26-LSL-Cas9-GFP* and we induced Cre recombination with 4-OHT at E8.5 (Figure 5A). This time, E8.5 was chosen as the selected time point of Cre induction instead of E7.5, as it results in higher recombination rates in FL c-Kit+ hematopoietic progenitors, including EMPs, but still largely avoiding the labeling of definitive-type adult HSCs, which emerge only after E10.5 ([17]; Barone et al., under revision). We next isolated CD45+ c-Kit+ GFP+ cells from E14.5 FL of *Cdh5-CreER^T2^::R26-LSL-Cas9-GFP* embryos by FACS (Figure 5B), and nucleofected them with sgRNAs against Mll and/or Af9, which, for consistency, we designed to target intronic breakpoints (intron 10 for Mll, intron 8 for Af9) analogous to the ones present in *Mll^loxP^::Af9^loxP^* mice (Figure 5C). When both sgRNAs were used together, we obtained clear evidence of the Mll-Af9 inter-chromosomal rearrangement by PCR (Figure 5D), which was also confirmed by Sanger sequencing (Figure 5E). The variability in the sequence of the Mll-Af9 rearrangement at the breakpoints reflects the fact that this analysis was conducted in bulk (Figure 5E). Next, after a 7-day co-culture period with OP9 stromal cells, we plated translocated and control non-transfected FL hematopoietic progenitors in a methylcellulose-based media containing cytokines. Already upon the first plating, edited cells gave rise to significantly more colonies than control cells, including large granulocyte, erythroid, monocyte, and megakaryocyte (GEMM) colonies, which were absent in control cells (Figure 5F). Mll-Af9 edited cells produced more colonies than control cells until the third round of replating, indicating that they had acquired an enhanced self-renewal potential (Figure 5F). Accordingly, the transplantation of Mll-Af9 edited FL HSPCs in sub-lethally irradiated recipients yielded a strongly myeloid-biased engraftment (Figure 5G). These data support the idea that Mll-Af9 confers enhanced self-renewal to HSC-independent myeloid progenitors.

## 4. Discussion

The mutations driving AML change dramatically with age [6]. Adult AML is mostly driven by the progressive accumulation of focal mutations, whereas the large majority of pediatric AML are characterized by the presence of fusion proteins originating from different structural variants [6,9]. Such diverse genetics are also reflected by the overall mutation burden, which is much higher in adult AML [9]. However, the real biological meaning of this phenomenon is still unclear. One hypothesis that has been proposed is that translocations could simply occur more frequently during embryonic/fetal hematopoiesis than during postnatal life [13]. This was supported by the idea that fetal HSCs cycle more frequently than adult HSCs, which are instead normally present in a more quiescent state [42]. Thus, the age-specific mutation profile would be consistent with the distinct type of DNA damage that HSPCs are exposed to at different ages in life. Fetal hematopoiesis would present mutations especially induced by replication stress, whereas adult life would expose HSPCs to the physiological age-driven accumulation of single nucleotide variants or indel mutations. Although this can certainly be a plausible explanation, it is probably not the only one.

Recent work shows that the pool of HSCs fated to adult life undergoes limited cell divisions during gestation [23]; thus, in this scenario, the previously observed extensive proliferation of FL HSPCs would primarily be carried out by a pool of stem/progenitor cells ultimately fated to differentiation [21,23,43]. If this relatively new understanding of fetal hematopoiesis eventually holds to be true, a possible consequence would imply that adult-fated HSCs are likely not the cell of origin of most hematological malignancies originating prenatally. Moreover, the higher frequency of chromosomal rearrangements would not be explained by replication stress-induced DNA damage, at least not in adult-fated HSCs.

The hypothesis that the intrinsic properties of fetal hematopoiesis may act as vulnerabilities for pediatric hematological malignancies is not a new one. Thus, another possible explanation for the age-specific mutation profile of AML could be that fetal myeloid progenitors are more susceptible than adult ones to leukemic transformation upon the acquisition of AML-associated translocations. Indeed, some investigators provided experimental support for this hypothesis. For example, the ETO2-GLIS2 fusion was shown to transform fetal progenitors with more efficiency than adult ones in mice [44]. In this model, the leukemic phenotype and latency were also ontogeny-dependent [44]. Therefore, developmental hematopoiesis would provide a “window of opportunity” for leukemic transformation, which could be largely driven by the specific cellular environment and high proliferation capacity of HSPCs distinct from adult-fated HSCs.

Among HSC-independent progenitors, EMPs were recently shown to substantially contribute to fetal erythropoiesis [19] as well as to long-lasting immune cells that, when subject to various perturbations, have the potential to affect adult disease [45]. So far, EMPs have not been recognized as potential cells of origin of pediatric AML, or other childhood myeloid neoplasms driven by inter-chromosomal translocations, probably due to their transiency and lack of long-lasting engraftment potential [46]. However, being intrinsically very proliferative [46], EMPs would remarkably fit the criteria of the higher susceptibility to replication stress damage.

Our work here shows that EMPs are, in principle, susceptible to Mll-Af9-mediated leukemic transformation. Although we have not formally determined the progenitor cell type undergoing malignant transformation in our experimental model, previous reports which we and others were able to reproduce have demonstrated that 4-OHT activation at E7.5 in the *Cdh5-CreER^T2^* model targets EMPs almost exclusively, avoiding recombination in the HSC-dependent wave [28,29]. Our lineage tracing experiments, presented in Figure 2, support the feasibility of using the *Cdh5-CreER^T2^* model to separately target EMP- and HSC-derived hematopoietic waves.

To our knowledge, this is the first report demonstrating that the selective introduction of a chromosomal rearrangement in HSC-independent progenitors can result in leukemic disease. A recent study employed a similar Cre-Lox translocator mice-based strategy to develop embryonic and adult models of Mll-Enl driven leukemia; however, the induction strategy in this study could not discriminate between HSC-dependent and independent waves [15]. Other reports conclusively reported a fetal origin for the Mll-Af4 pro-B acute lymphoblastic leukemia, one of the most common hematological malignancies in infants [47,48]. The fetal lymphoid-primed multipotent progenitor (LMPP) [49] was suggested as a potential cell of origin [50], although the experimental models used were not able to discriminate between HSC-dependent or independent hematopoietic waves. Another study reported T-ALL development upon the overexpression of the Notch intracellular domain in early intraembryonic, but not yolk sac, precursors [51]. Once again, this study did not explain what the precise nature of leukemia propagating cells was in vivo, although the fact that only intraembryonic cells were susceptible to leukemic transformation in this setting was a strong indicator of an HSC origin, in agreement with EMPs being independent of Notch signaling [52,53]. Overall, these studies provide a strong experimental rationale supporting that several types of infant leukemias can have a fetal origin, but, at the same time, highlight the difficulty of identifying which progenitor cell types are susceptible to transformation.

One important characteristic of the experimental models we used in this study is that both the Cre-Lox model and the CRISPR/Cas9-based approach rely on inter-chromosomal translocation events; therefore, the expression of deriving fusion genes is driven by endogenous regulatory elements rather than by an extrinsic promoter, making the model more physiological than transgene overexpression. A consequential limitation is that the efficiency of leukemic transformation was probably low, and this was reflected by the long incubation times we observed. Additionally, in the Cre-Lox model, not all cells in which the Cre is expressed will generate an inter-chromosomal translocation. Our conditional Cre activation protocol induced recombination in a subset of cells in a short time window during embryonic development; therefore, it is highly probable that when using a conditional strategy, the clones in which the Mll-Af9 translocation is generated are of a small size. Indeed, except in rare cases, a clear leukemic phenotype was seen only in transplanted mice, possibly because lethal irradiation acted as a perturbation trigger that allowed transformed clones to expand.

Our data suggest that in infant AML, the acquisition of a myeloid-biased self-renewal program driven by Mll-Af9 in HSC-independent progenitors (EMPs) that are normally highly proliferating could lead to leukemic transformation. Interestingly, a myeloid phenotype was only observed when Mll-Af9 was introduced in EMPs, but not in embryonic HSCs, at least within the timeframe of our analysis. Our results are consistent with the delayed latency and mixed phenotype observed when Mll-Af9 was introduced in fetal HSCs using a knockin model [14]. Nevertheless, our results do not exclude that HSCs could also act as cells of origin for infant AML; however, it reinforces the possibility that differences in the cell of origin could influence the latency and the phenotype of the disease.

In our experimental setting, we could not determine whether embryonic-derived Mll-Af9 leukemia was more aggressive than its adult counterpart because all our experiments were performed in the context of embryonic hematopoiesis. Instead, we focused on determining which of the embryonic hematopoietic waves contained cells susceptible to leukemic transformation upon the introduction of Mll-Af9 rearrangement. Besides the proliferation rate, what other specific characteristics of HSPCs belonging to distinct embryonic hematopoietic waves would make them more or less vulnerable to leukemic transformation remains an open question. Recent studies reported a window of opportunity for leukemogenesis in neonatal blood progenitors upon the introduction of the Mll-Enl translocation [16]. The same group also reported that the transition to fetal and adult hematopoiesis taking place in neonates was accompanied by a peak in IFN signaling [42]. Intriguingly, pro-inflammatory signaling, including IFNs, was also shown to be particularly important for embryonic HSC development [54]. It will be interesting to further explore the role of inflammation in pediatric leukemias, especially in the context of recent insight in developmental hematopoiesis.

## 5. Conclusions

In conclusion, here we provide an experimental “proof-of-principle” showing that HSC-independent hematopoietic progenitors, which include EMPs, are susceptible to leukemogenesis upon the acquisition of the Mll-Af9 inter-chromosomal rearrangement. Further studies should investigate the specific intrinsic and extrinsic characteristics of the cellular environment in embryonic and fetal hematopoiesis, and the molecular pathways that might be responsible for malignant transformation. Obtaining such insights will be important in order to identify the potentially targetable and fetal-specific vulnerabilities of infant leukemias.

## Data Availability

The data presented in this study are available on request from the corresponding author.

## Supplementary Materials

The following supporting information can be downloaded at https://www.mdpi.com/article/10.3390/cancers15143624/s1, Figure S1: Additional analysis of *Tie2-Cre::Mll^loxP^::Af9^loxP^* mice and transplants; Figure S2: Analysis of unperturbed adult *Cdh5-CreER^T2^*::*Mll^loxP^::Af9^loxP^* mice; Figure S3: Detection of Mll-Af9 translocation in *Cdh5-CreER^T2^*::*Mll^loxP^::Af9^loxP^* transplants; Table S1: Primer sequences used for Mll-Af9 detection by PCR.
