# Supplementary material for: Hematopoietic Stem Cell (HSC)-Independent Progenitors Are Susceptible to Mll-Af9-Induced Leukemic Transformation
Source: Cancers (Basel). 2023 Jul 14;15(14):3624. doi: 10.3390/cancers15143624 (PMC10377085; doi:10.3390/cancers15143624)
Supplement: Supplementary file 1 [file cancers-15-03624-s001.zip › cancers-2439998-supplementary.pdf]

Supplementary Figures and Legends

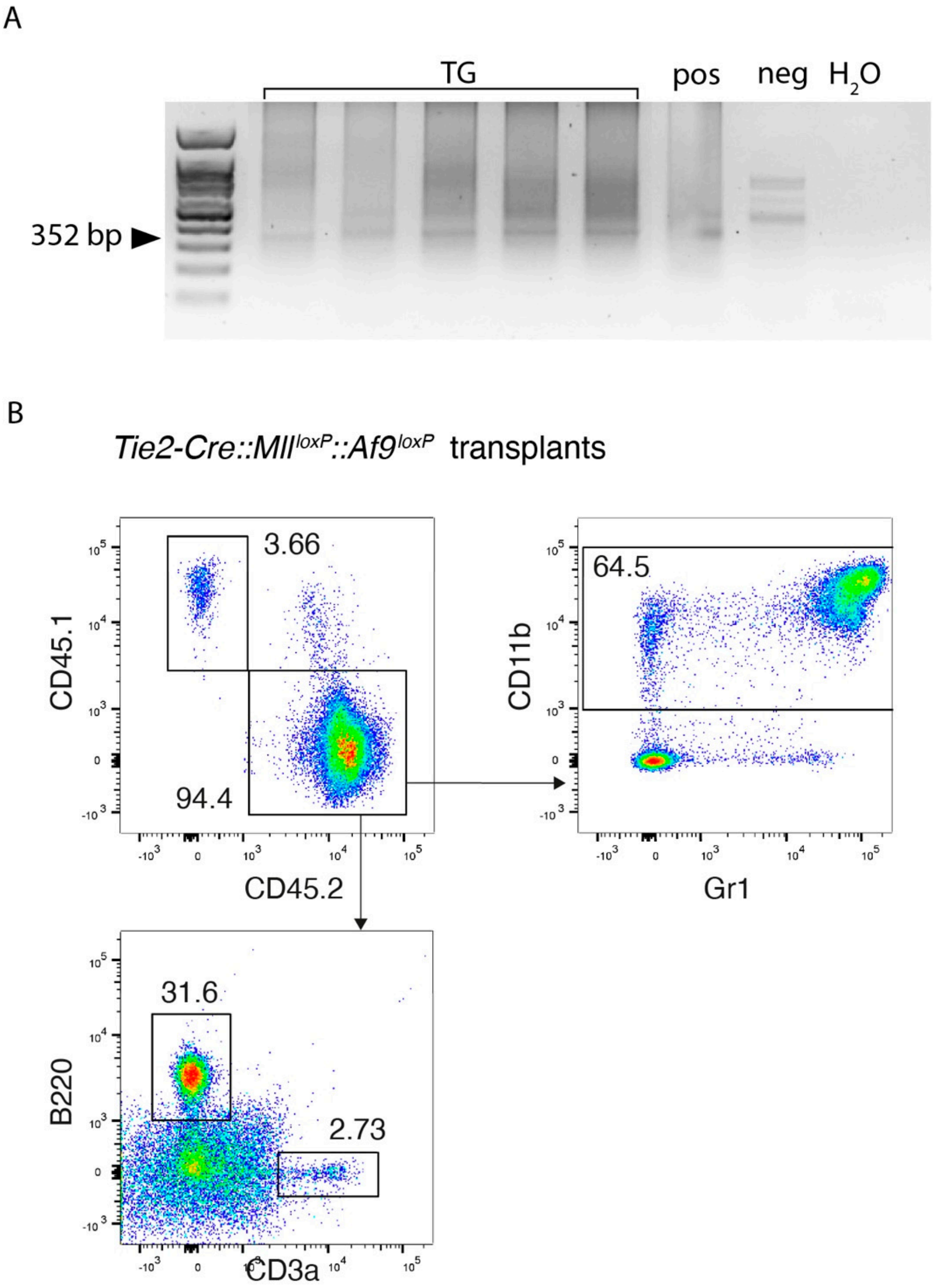

Figure S1. Additional analysis of *Tie2-Cre::Mll^{loxP}::Af9^{loxP}* mice and transplants.

(A) Agarose gel showing the presence of the expected size amplicon (black arrow head) corresponding to the Mll-Af9 translocation in the bone marrow (BM) of *Tie2-Cre::Mll^{loxP}::Af9^{loxP}* mice (TG). Positive (pos) and negative (neg) controls are shown.

(B) Representative flow cytometric peripheral blood (PB) analysis of lethally irradiated mice transplanted with BM cells derived from *Tie2-Cre::Mll^{loxP}::Af9^{loxP}* adult mice. Donor-derived chimerism (CD45.1 vs CD45.2, top left), myeloid cells (CD11b vs Gr1, top right), B and T lymphocytes (B220 vs CD3a, bottom) were assessed.

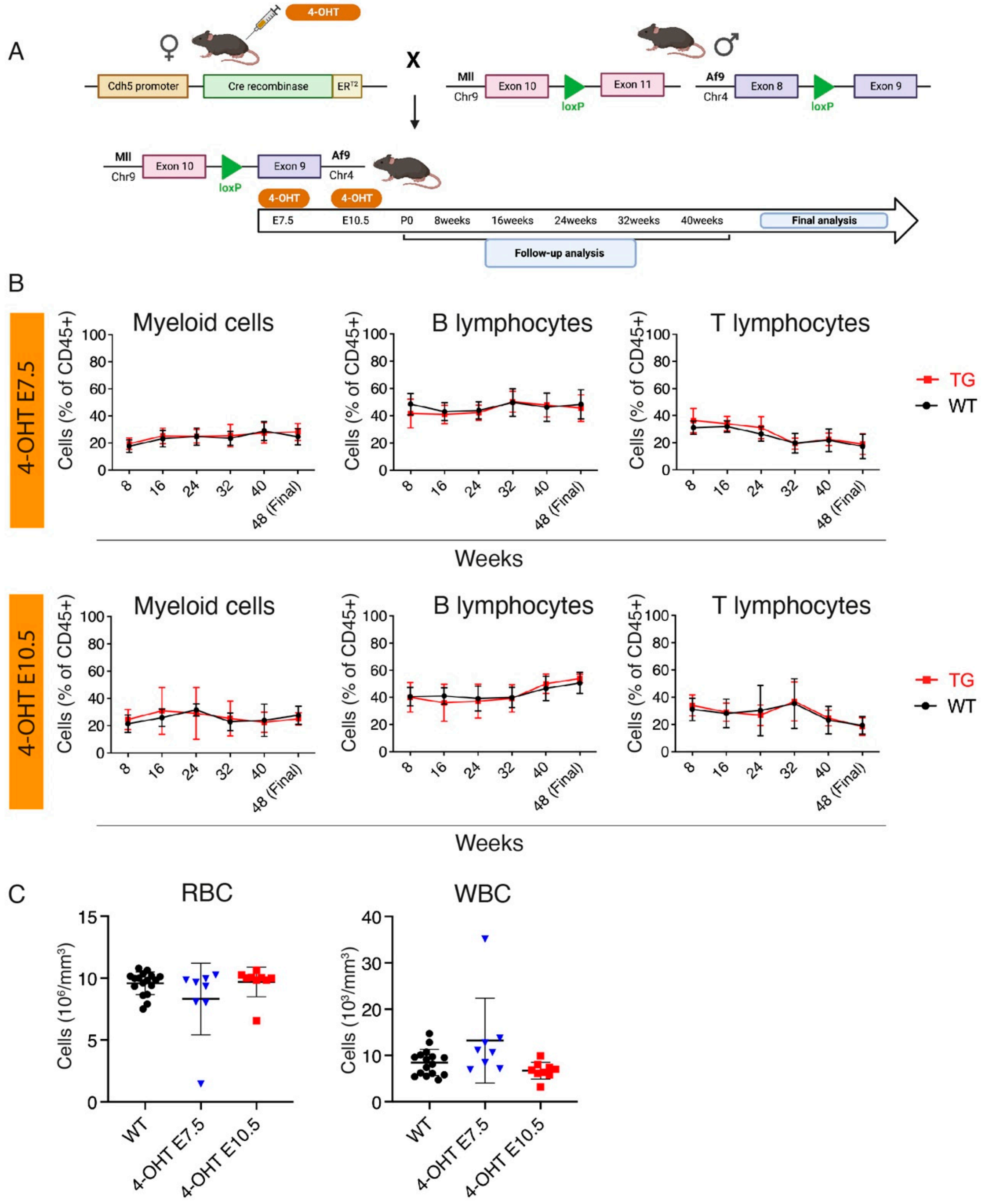

Figure S2. Analysis of unperturbed adult *Cdh5-CreER^{T2}::Mll^{loxP}::Af9^{loxP}* mice.

(A) Schematic of crossing strategy, Cre activation (4-OHT at E7.5 or E10.5) and analyses of adult *Cdh5-CreER^{T2}::Mll^{loxP}::Af9^{loxP}* mice.
(B) Quantification of flow cytometric analysis of myeloid (left), B (middle) and T lymphocytes (right) in WT (black) and *Cdh5-CreER^{T2}::Mll^{loxP}::Af9^{loxP}* (TG, red) unperturbed adult mice PB, with the administration of 4-OHT at E7.5 (top) or E10.5 (bottom). Data are mean ± SD.
(C) Red blood cells (RBC, left) and white blood cells (WBC, right) absolute counts of 48 weeks-old WT (black) and *Cdh5-CreER^{T2}::Mll^{loxP}::Af9^{loxP}* adult mice with the administration of 4-OHT at E7.5 (blue) or E10.5 (red). Data are mean ± SD.

A

	FL		BM primary tx		BM secondary tx		Fraction of Mll-AF9 pos mice
	Mll-AF9 positive	Total	Mll-AF9 positive	Total	Mll-AF9 positive	Total	
Tx 4-OHT E7.5	6	6	6	6	5	8	**17/20 (85%)**
Tx Tam E10.5	4	8	2	8	4	5	**10/21 (47.6%)**

B

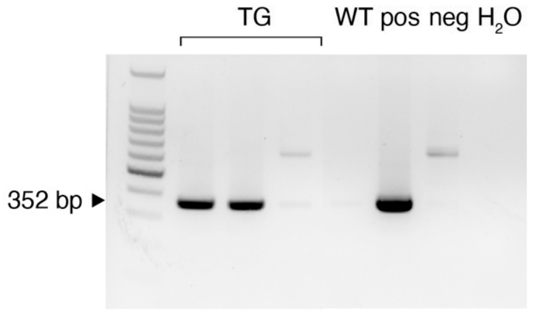

Figure S3. Detection of Mll-Af9 translocation in *Cdh5-CreER^{T2}::Mll^{loxP}::Af9^{loxP}* transplants.

(A) Table representing the number of samples, per condition and total, used for primary and secondary transplantation analysis of *Cdh5-CreER^{T2}::Mll^{loxP}::Af9^{loxP}* (4-OHT at E7.5 or Tamoxifen at E10.5) FL and BM. This table summarizes the number of samples in which the Mll-Af9 translocation was detected by PCR. The “BM primary tx” and “BM secondary tx” columns refer to the analysis done on the BM of transplant recipients.
(B) Representative agarose gel showing the presence of the expected 352-bp amplicon corresponding to the Mll-Af9 inter-chromosomal translocation in 2/3 *Cdh5-CreER^{T2}::Mll^{loxP}::Af9^{loxP}* transgenic (TG) FL samples (Tamoxifen at E10.5). Positive control (pos), wild type (WT) and negative (neg) controls are shown.

Primer name	Sequence (5'-3')	Cre/CRISPR
1A_Mll-intron_FW	GTC CCC ATA ACA CCC AGA GTA GTG	Cre
1B_AF9-intron_Rev	CCT CAT TCT GAC AGA CCA GAG CCA	Cre
2A_Mll-intron_FW	GGG CAT GTA GAG GTA AGA CGC CTG	Cre
2B_AF9-intron_Rev	ATC TCC AGG GAC TGA ATC TAG GGC	Cre
Mll_int10-CRISPR_FW	AAA CCA ACA GCA ACC CTT TTT	CRISPR
Mll_int10-CRISPR_Rev	GCA GTG GGC ATG TAG AGG TAA	CRISPR
AF9_int8-CRISPR_FW	TTT GTT CCC ATC ACA TCT GCC	CRISPR
AF9_int8-CRISPR_Rev	GGC AGC CAC TCA GTA ACT TG	CRISPR

Table S1. Primer sequences used for Mll-Af9 detection by PCR.
The third column indicates if primers were used to detect Cre/LoxP-induced or CRISPR/Cas9-induced translocation.
